# Automated equilibrium tension lysimeter deployment to monitor vadose zone soil water and solute transport

**DOI:** 10.1002/jeq2.70272

**Published:** 2026-10-06

**Authors:** Mitch D. J. Wodrich, Shuohao Cai, Kevin C. Masarik, Jingyi Huang, Matthew D. Ruark, Xia Zhu‐Barker

**Affiliations:** ^1^ Department of Soil and Environmental Sciences University of Wisconsin‐Madison Madison Wisconsin USA; ^2^ College of Natural Resources University of Wisconsin‐Stevens Point Stevens Point Wisconsin USA

## Abstract

Accurate quantification of deep percolation is fundamental for understanding vadose zone hydrology, groundwater recharge, and solute transport in agricultural landscapes. Many lysimeter designs impose fixed or passively generated suction that does not continuously respond to changes in the surrounding soil hydraulic environment, limiting the conditions under which representative drainage is collected. Automated equilibrium tension lysimeters (AETLs) aim to address this limitation by continuously adjusting lysimeter suction to match bulk soil matric potential. However, existing AETL systems are constrained by high power demands, limited sensor performance, and complex operation. This paper presents an updated AETL system that integrates contemporary hardware, solar‐powered operation, and remote communication to improve system reliability and temporal resolution. Sixteen AETLs installed at 120‐cm depth in silt loam soils in south‐central Wisconsin were monitored during the 2025 growing season. Performance evaluation indicated that several systems were not fully equilibrated during early spring, suggesting drainage may have been under‐collected. Despite this limitation, drainage occurred primarily during late winter and spring when soils approached saturation, whereas summer precipitation rarely produced drainage. Drainage varied substantially among lysimeters (∼1%–40% of cumulative precipitation), reflecting early‐season operational variability and microtopographic controls on water redistribution, contextualized using topographic wetness index. An independent soil‐water budget showed drainage from better performing lysimeters fell within the expected range, while drainage from the poorer performing lysimeters did not. The updated AETL system enables high‐resolution monitoring of deep percolation in non‐tile‐drained agricultural systems, though performance remains constrained by the power and hydraulic‐contact limitations documented here.

## Core Ideas

- Reliable deep drainage measurement remains underdeveloped in non‐tile‐drained agricultural soils.
- The updated AETL system enables continuous, high‐resolution drainage measurement with documented operational tradeoffs.
- Deep drainage is episodic and spatially variable, requiring long‐term, plot‐scale monitoring.

## Plain Language Summary

Water moving below plant roots carries nutrients and other chemicals toward groundwater, but this process is difficult to measure accurately in farm fields. Many existing tools can misrepresent how much water actually drains through soil. In this study, we tested an automated lysimeter network designed to track water movement by adjusting to changing soil moisture conditions. We monitored 16 lysimeters in a Wisconsin field and found that most drainage occurred during late winter and spring when soils were wet, while summer rainfall produced little deep drainage. We also observed large differences in drainage between lysimeters, possibly due to system performance and variations in landscape position. These results show that improved monitoring systems can help better understand when and where water and nutrients move below the root zone.

**Abbreviations**
AETL: automated equilibrium tension lysimeter
ET: evapotranspiration
PFAS: per‐ and polyfluoroalkyl substances
TWI: topographic wetness index

## 1 INTRODUCTION

Accurate quantification of the water flux below the root zone is fundamental to understanding vadose zone hydrology, groundwater recharge, and solute transport in agricultural landscapes. Deep percolation governs the movement of dissolved solutes, including inorganic nutrients, dissolved organic carbon and nitrogen, per‐ and polyfluoroalkyl substances, pesticides, and other agrochemicals, from soils to underlying groundwater. However, drainage below the root zone is neither continuous nor spatially uniform, and is strongly influenced by soil hydraulic properties, profile stratification, and climatic variability (Scanlon et al., 2002). Because solute transport is intrinsically linked to the timing and magnitude of water movement through soil, reliable measurement of deep percolation is essential for understanding both hydrologic processes and subsurface solute transport.

A central challenge in quantifying drainage is its episodic nature. Numerous studies in the Upper US Midwest have demonstrated that a large proportion of annual drainage occurs during short seasonal windows, typically in late winter and early spring when precipitation or snowmelt inputs are large, evapotranspiration (ET) is low, and soils are near saturation (Brye et al., 2000; Masarik et al., 2014; Stenjem et al., 2019; Syswerda et al., 2012). Outside of these periods, deep percolation can be limited even after large precipitation events, particularly where discontinuities in soil texture create vertical flow restrictions (Li et al., 2017; Ross, 1990).

In many agricultural regions, subsurface tile drainage provides a practical means of intercepting drainage water and assessing subsurface transport. However, tile drainage captures only a fraction of percolating water and reflects flow pathways that are strongly influenced by drainage design, spacing, depth, and hydraulic connectivity rather than the full vertical flux through the soil profile (Boles et al., 2015; Dinnes et al., 2002). Tile installation also alters soil structure and preferential flow pathways near the drain, making effluent measurements difficult to scale to whole‐plot drainage (Skaggs et al., 1994). Moreover, subsurface tile drainage is uncommon across large portions of the Upper US Midwest, necessitating alternative approaches for quantifying water fluxes and solute transport below the root zone in non‐tile‐drained landscapes (United States Department of Agriculture, 2024; Wan et al., 2024). In situ lysimetry offers an alternative by characterizing drainage under field conditions that more closely represent natural soil water movement.

A variety of lysimeter designs have been used to estimate soil water fluxes and solute leaching, each with inherent advantages and limitations. Zero‐tension and fixed‐tension lysimeters are relatively inexpensive options for measuring drainage and solute fluxes; however, they are prone to under‐ or overestimating drainage because they impose a static suction regime that does not continuously reflect surrounding soil matric potentials or the hydraulic gradient. Specifically, drainage enters zero‐tension lysimeters only under saturated conditions, whereas fixed‐tension lysimeters may over collect during wet periods when applied suction exceeds the matric potential (Brye et al., 1999). Under drier conditions, insufficient suction may cause water to diverge around the lysimeters, underestimating drainage and solute fluxes (Jemison & Fox, 1992; Radulovich & Sollins, 1987).

Passive capillary wick samplers represent a distinct class of tension lysimeter that partially addresses these limitations. An inert fiberglass wick forms a hanging water column that applies suction to the overlying soil and draws drainage without pumps or active pressure control (Boll et al., 1992; Knutson & Selker, 1994). Because wick‐generated suction varies with drainage flux, rather than remaining fixed, a properly designed sampler can approximate the matric potential of the surrounding soil across a range of fluxes. Wick samplers also offer practical advantages, including passive operation, low power requirements, minimal electronic complexity, and demonstrated durability over multiyear field deployments (Feaga et al., 2010; Gee et al., 2009; Louie et al., 2000). However, the suction generated by a hanging water column is generally limited to approximately −50 to −100 cm of water (roughly −5 to −10 kPa), and matching the more negative matric potentials common in fine‐textured soils requires impractically long wicks (Boll et al., 1992; Knutson & Selker, 1994). Consequently, both modeling and field evaluations have shown that wick sampler collection efficiency declines in fine‐textured soils, particularly under low drainage fluxes that promote flow divergence around the sampler (Gee et al., 2009). Such conditions are common in many non‐tile‐drained agricultural soils in the Upper US Midwest.

Equilibrium tension lysimeters were developed to more closely reproduce the hydraulic conditions of the surrounding soil (Brye et al., 1999). Suction at the collection surface is adjusted to match the matric potential of the adjacent soil, minimizing disturbance to the natural hydraulic gradient and improving estimates of drainage and percolation fluxes. Traditional equilibrium systems, however, require frequent, manual adjustment based on concurrent measurements of soil matric potential, increasing labor demands and limiting temporal resolution (Brye et al., 1999; Masarik et al., 2004). Automated equilibrium tension lysimeters (AETLs) address these constraints by automating control of lysimeter suction in response to real‐time measurements of soil matric potential (Masarik et al., 2004). By minimizing deviations between applied suction and surrounding soil matric potential, AETLs can provide temporally resolved measurements of deep percolation and solute leaching under hydraulic conditions that more closely approximate the undisturbed soil profile (Farsad et al., 2019; Masarik et al., 2004, 2014).

Unlike passive wick lysimeters, AETLs generate suction through active vacuum control and can therefore match substantially more negative matric potentials. This capacity makes them well suited for the fine‐textured, low‐flux conditions common in non‐tile‐drained Upper US Midwestern agroecosystems. Active control, however, introduces a different set of tradeoffs. AETL systems are technically complex and depend on continuous power to regulate suction (Masarik et al., 2004). Performance can also degrade under dry soil conditions, when maintaining hydraulic continuity between the lysimeter and surrounding soil becomes difficult (Brye et al., 1999).

This paper describes the design, deployment, and field performance of an updated AETL system adapted from Brye et al. (1999) and Masarik et al. (2004). The system incorporates contemporary dataloggers and relay drivers, improved matric potential sensors, solar recharge, and cellular and radio communications for remote monitoring and control. Documented here are key design considerations and operational challenges, including the interaction between sustained pumping under dry soil conditions and solar recharge, and the control‐logic modifications implemented in response. One growing season of measurements illustrates system performance and seasonal patterns in deep soil water fluxes. While the AETL system is designed to support solute transport studies, solute analyses were beyond the scope of this manuscript and will be addressed in future work.

**Core Ideas**

- Reliable deep drainage measurement remains underdeveloped in non‐tile‐drained agricultural soils.
- The updated AETL system enables continuous, high‐resolution drainage measurement with documented operational tradeoffs.
- Deep drainage is episodic and spatially variable, requiring long‐term, plot‐scale monitoring.

## 2 METHODS

### 2.1 Study site

The study was conducted at the University of Wisconsin Arlington Agricultural Research Station in Arlington, Wisconsin (Figure 1). Based on 30‐year climate normals, average annual precipitation at the site is 88.6 cm and average annual air temperature is 7.8°C (Midwestern Regional Climate Center. Purdue University, 2026). The study area is dominated by Ringwood and Plano silt loam soils, classified as fine‐loamy and fine‐silty, mixed, superactive, mesic Typic Arguidolls, respectively. These soils are characterized by a fine‐textured Bt horizon overlying a coarse‐textured C horizon, with the Bt‐C transition occurring at depths of approximately 91–117 cm (Soil Survey Staff, 2026). The soil parent materials are loess over glacial loamy till (Soil Survey Staff, 2026).

**FIGURE 1:**
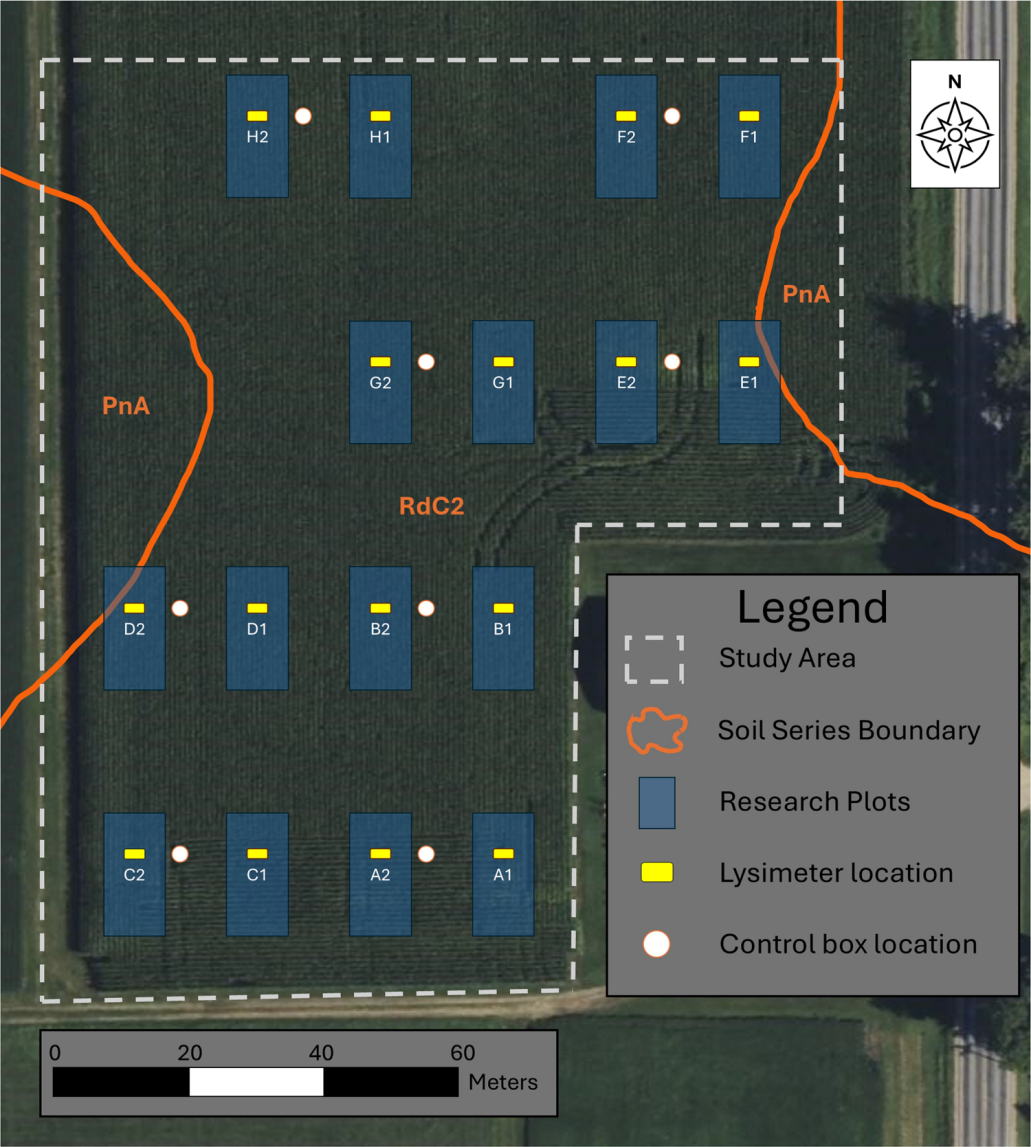
Map of study site with plot labels. The following soils are present in the study area: RdC2: Ringwood silt loam, 6%–12% slopes; PnA: Plano silt loam, 0%–2% slopes. Lysimeter and control box size are not to scale.

### 2.2 AETL installation

Sixteen plots, each approximately 9.1 m × 18.3 m, were established across the study area, with one AETL installed in each plot (Figure 1). Lysimeters were positioned approximately 4.6 m from the eastern plot boundary and 6.1 m from the northern boundary to minimize potential edge effects. Installation was completed in Fall 2023, and drainage collection commenced in Summer 2024. One control system operated two lysimeters, resulting in a total of eight control systems and 16 AETLs distributed across the site (Figure 1).

All lysimeters were cleaned and constructed following the specifications described by Brye et al. (1999), with minor modifications to the construction described below (Figure 2). Lysimeter dimensions were 76.2 cm wide × 15.2 cm tall × 25.4 cm deep resulting in a 0.194‐m^2^ collection area. A rectangular frame (5.5 cm tall) was spot welded around the lysimeter such that 0.63 cm (1/4 in) extended above the stainless steel porous collection plate (316L SS, 0.2‐µm pore size, 0.039 in thickness, liquid permeability coefficient *K_L_* = 90; Mott Corporation), creating a lip. This frame differed from the 10.2‐cm frame with a 2.5‐cm‐tall lip in the Brye et al. (1999) construction. The seams between the frame and the stainless steel lysimeter sides were sealed with stainless steel putty (10270; Devcon). Additionally, eight arched‐steel support braces were welded inside the lysimeter to provide structural support (Figure 2). The braces were positioned with roughly 8.4‐cm spacing.

**FIGURE 2:**
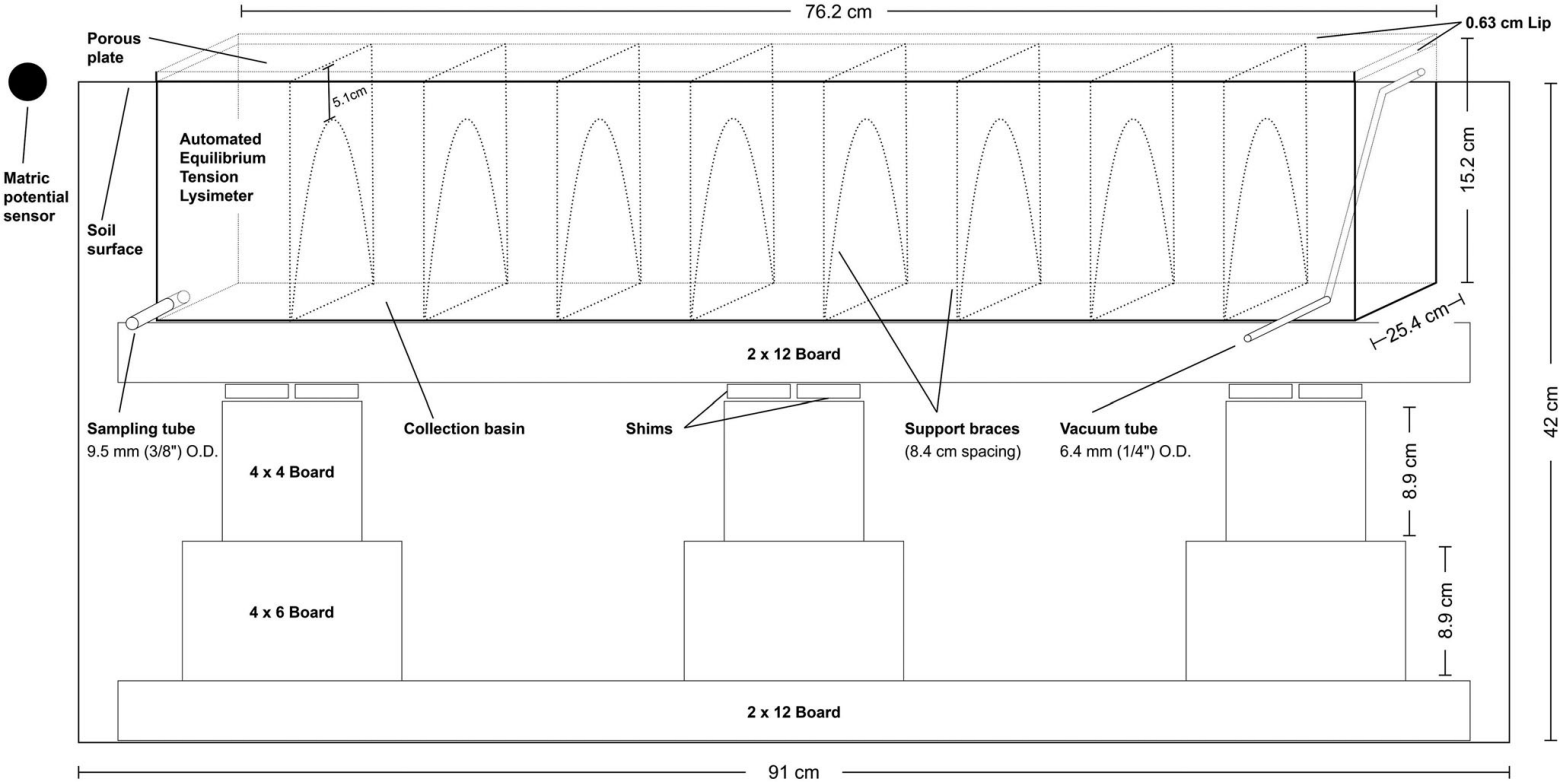
Diagram of the features, dimensions, and installation of the automated equilibrium tension lysimeter in the soil cavity.

Installation involved excavating a trench approximately 100 cm wide and 200 cm deep, followed by digging a cavity in the trench sidewall measuring 91 cm wide × 42 cm tall × 40 cm deep. The base of the cavity was approximately 162 cm below the soil surface, allowing the top of the lysimeter to be positioned at a depth of 120 cm. This depth was selected to be below the primary root zone of corn (*Zea mays L*.) and representative of drainage bound for groundwater. A pressure‐treated 3.8 cm thick × 28.6 cm wide × approximately 90 cm long (nominal 2 by 12 in) wooden board was placed at the base of the cavity to provide structural support. The lysimeter was positioned at the top of the cavity with an equally sized board below the lysimeter. Both were raised using scissor jacks until the porous collection plate contacted the undisturbed soil column above. The lysimeter was oriented with a slight lateral tilt to facilitate drainage collection toward the sampling port. Pressure‐treated wooden blocks, positioned with wood grain vertically, were wedged between the support board and the lysimeter base board to secure the lysimeter's position. If necessary, wooden shims were also added before the scissor jacks were removed. This approach preserved the undisturbed soil column above the lysimeter.

Brye et al. (1999) originally used screw jacks and spring plates to maintain contact between the porous collection plate and the soil. However, when failed AETLs from that study were excavated in 2006, the spring plates were found to be fully compressed and no longer capable of maintaining continuous soil‐lysimeter contact (Lepore et al., 2011). Pressure‐treated wooden blocks were therefore installed with grain oriented vertically, providing greater resistance to compressive forces. Matric potential sensors (Teros 21 Gen 2; Meter Group) were installed adjacent to each lysimeter at a depth of 120 cm to support equilibrium tension control during drainage collection.

### 2.3 AETL control system

Each control system was designed to operate two AETLs. A schematic of the electrical and pneumatic components is shown in Figure 3. The control logic described by Masarik et al. (2004) served as the foundation for the system design, with modifications made to incorporate contemporary hardware and additional features.

**FIGURE 3:**
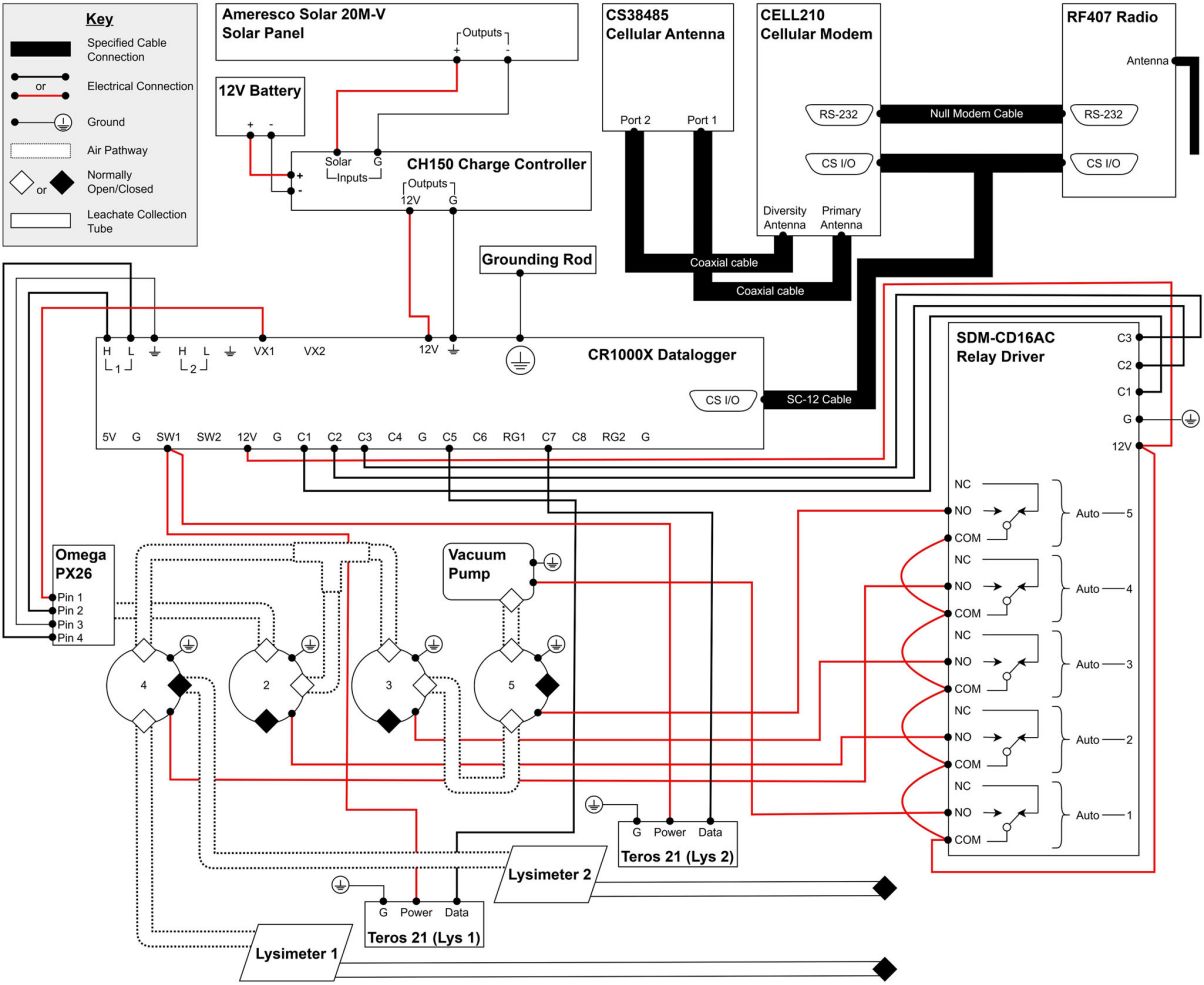
Electrical and pneumatic schematic of the automated equilibrium tension lysimeter system.

Soil matric potential at a depth of 120 cm, measured by Teros 21 Gen 2 matric potential sensors (Meter Group), was used to control lysimeter suction. These sensors provided an improved measurement range and were factory calibrated for matric potentials between 0 and −100 kPa with an accuracy of ± (10% of reading + 2 kPa). Factory calibration includes measurements at saturation (0 kPa), at −100,000 kPa, and at four points between 0 and −100 kPa. No additional calibration, drift checks, or post‐processing was applied beyond factory calibration. To support autonomous field operation, each control system was equipped with a 20‐W solar panel (20 M; Ameresco Solar) and a charge controller (CH150; Campbell Scientific) to provide battery recharge and reduce the need for manual recharging. One of the eight control systems was outfitted with a cellular modem (CELL210; Campbell Scientific) and antenna (Model 38485; Campbell Scientific) to provide internet connectivity. Data transfer between individual (“leaf”) dataloggers and the centrally connected datalogger was facilitated using spread‐spectrum radios (RF407; Campbell Scientific) installed on each control system. Remote system access enabled timely data retrieval and allowed software updates to be deployed without frequent site visits.

Each AETL was controlled by a CR1000X datalogger (Campbell Scientific) and powered by a 12‐V, 8 A‐h, lead‐acid battery charged via the solar array. Lysimeter suction was monitored using a piezoresistive gauge pressure transducer (PX26‐005GV; Omega Engineering), with bridge excitation supplied by the datalogger. To correct for transducer zero drift, the lysimeter chamber was vented to atmosphere immediately prior to each pressure measurement and an atmospheric reference reading was recorded; chamber suction was then computed as the difference between this reference and the subsequent closed‐chamber reading. This in situ zeroing was performed on every measurement cycle, such that transducer offset drift was continuously corrected. Transducer span was applied using the nominal transfer function; individual span calibration was not performed during this deployment. Suction was increased using a vacuum pump (TD‐2N; Brailsford & Co.) or decreased by venting the lysimeter to the atmosphere until equilibrium with the measured soil matric potential was approximated. Four, three‐way, normally open, solenoid valves (ETO‐3‐12 and 11752–3; Clippard Instrument Laboratory) were connected to the vacuum pump and pressure transducer using clear flexible PVC tubing (14‐387‐338; Thermo Fisher Scientific). A 12‐V relay driver (SDM‐CD16AC; Campbell Scientific) controlled the operation of the pump and solenoid valves.

All control system components were housed within a fiberglass enclosure (ENC 16/18; Campbell Scientific). Vacuum and sampling lines were routed from each lysimeter to the enclosure through underground PVC conduit; component specifications and tubing dimensions are provided in Supporting Information S1.

The CR1000X dataloggers executed a control program adapted from the logic described by Masarik et al. (2004) to maintain lysimeter suction in equilibrium with surrounding soil matric potential. Program scans were performed every 10 min. During each scan, the soil matric potential adjacent to each lysimeter was measured, and a target suction was calculated for each lysimeter. The remaining scan time was divided evenly between the two lysimeters, with approximately 5 min allocated per lysimeter to either increase or decrease suction to reach the target value. A suction offset between 2 and 5 kPa greater than the measured soil matric potential was applied to establish a small hydraulic gradient to drive drainage into the lysimeter, consistent with previous implementations (Brye et al., 1999; Masarik et al., 2004). Maximum lysimeter suction was capped at −35 kPa, slightly stronger than a conservative estimate of field capacity (typically between −10 kPa to −33 kPa), beyond which vertical water movement and solute transport is minimal. Hourly averages were recorded for lysimeter suction, adjacent soil matric potential and soil temperature (120‐cm depth), temperature within the datalogger enclosure, and battery voltage.

### 2.4 Crop management

Grain corn was grown in 2024 and managed according to University of Wisconsin‐Extension recommendations for nutrient and pest management (Dewerff et al., 2024; Laboksi & Peters, 2012). Prior to planting in 2024, the site was disk chiseled to a depth of approximately 20 cm and subsequently cultivated to a depth of approximately 10 cm. In 2024, pre‐plant fertilizer (31‐0‐15‐3S) was applied at a rate of 448 kg ha^−1^, Dekalb DKC48‐34RIB corn was mechanically planted within plots at a rate of 89,000 seeds ha^−1^, and a Lacrosse Waterway Mix was established between plots at a rate of 28 kg ha^−1^. In 2025, urea (46‐0‐0) was applied pre‐plant at a rate of 482 kg ha^−1^, and Pioneer P0924Q corn was planted at a rate of 89,000 seeds ha^−1^ and grown for silage. Selective herbicides (Buc 5 Extra, Sharpen, and Strellius II) were applied pre‐emergence following label recommendations, with management following the same UW‐Extension guidelines.

### 2.5 Sample collection and analyses

Drainage collection began in June 2024; however, data collected prior to March 2025 are not presented here, as this initial period was used for system equilibration, installation disturbance recovery, and calibration of datalogger control systems. This approach allowed soil structure to reestablish and minimized the influence of artificial flow pathways created during lysimeter installation. Drainage was collected from each lysimeter on a biweekly basis during the non‐winter months (March–November) and monthly during the winter months (December–February). Drainage samples were extracted from the lysimeter sampling ports using a peristaltic pump (Model 410; Solinst). To ensure that samples reflected current lysimeter drainage, the first 100 mL were discarded to flush the sampling tubing. Subsequently, up to 1 L of drainage was collected and transported to the laboratory, where samples were filtered through 0.3‐µm glass fiber filters (Advantec GF‐75) and stored at −20°C until analysis. Any remaining drainage exceeding 1.1 L was measured in the field to quantify total drainage and then discarded.

Data recorded by the dataloggers were retrieved at the same frequency as drainage collection. Data were downloaded via wired connection until remote retrieval was established in April 2025. As described in Section 3.2, intermittent power losses occurred for some control systems, resulting in periods during which data were not recorded. Consequently, gaps in the time series may be evident for individual lysimeters. Precipitation data were retrieved from the Arlington, WI station of Wisconet, and Wisconsin's Environmental Mesonet (University of Wisconsin‐Madison, 2025).

The topographic wetness index (TWI) was calculated for each plot pair (A‐H) using Wisconsin high‐resolution digital elevation model (3‐meter resolution) data (Wisconsin Department of Natural Resources, 2019) based on the following formula:

(1) $$\mathrm{TWI}=\ln\left[\;\frac{a}{\tan\left(b\right)}\right]$$

where *a* and *b* are upslope contributing area (m^2^) and slope (radians), respectively. The TWI is a reference to estimate where water will accumulate in an area with elevation differences.

### 2.6 Data processing and visualization

Precipitation, soil matric potential, lysimeter suction, and drainage volume data were quality checked and plotted as time series without statistical analysis. These data are intended to demonstrate system performance and behavior during the first operational season rather than to support inferential comparisons among plots; spatial patterns in drainage collection are therefore described qualitatively and interpreted as preliminary results. Data gaps resulting from power loss or system downtime were retained to accurately reflect system performance.

### 2.7 Soil‐water budget

To provide an independent plausibility check on the drainage volumes measured by the AETL network, a simple soil‐water budget was calculated for the monitoring period (March–October 2025). Daily reference evapotranspiration (ETo) was also obtained from the Arlington, WI Wisconet station (University of Wisconsin‐Madison, 2025). Actual evapotranspiration (ETa) was estimated following the FAO‐56 single‐coefficient approach (Allen et al., 1998) using the following formula:

(2) ETa=ETo×Kc×Ks

where Kc is a crop coefficient and Ks is a soil‐water stress coefficient. The crop coefficient was derived from Sentinel‐2 normalized difference vegetation index (NDVI) using the following empirical relationship (Belmonte et al., 2005; D'Urso, 2010):

(3) Kc=1.25×NDVI+0.2

Since both the stress coefficient (Ks) and the crop coefficient (Kc) are approximations, and because their associated uncertainties act on ETa in opposing directions, the budget was evaluated under three parameterizations of the stress coefficient (Ks) to bracket the plausible range of drainage: (i) Ks scaled between the observed minimum and maximum soil moisture, treating these as wilting point and field capacity; (ii) Ks fixed at 1, representing an unstressed upper bound on ETa; and (iii) Ks scaled using a wilting point of 0.15 m^3^ m^−3^, more representative of the silt loam soils at the site, with field capacity set to the observed maximum. This analysis is intended as a bracketing consistency check rather than a calibrated drainage estimate. Field‐specific soil moisture spanning the monitoring period was not available, precluding an explicit soil‐water storage term. Storage change and surface runoff, which were not quantified, may shift the estimates further or contribute to observed variability among individual lysimeters.

Prior to corn planting (April 30, 2025), the field consisted of bare soil with previous season crop residue. Wisconet station soil moisture was measured at depths of 5, 10, 20, 51, and 102 cm; values were depth‐weighted across these measurements, and soil moisture from 102 to 120 cm was estimated using data from the deepest sensor. Drainage was then estimated as:

(4) Estimateddrainage=Precipitation−ETa

## 3 RESULTS AND DISCUSSION

### 3.1 Precipitation, soil matric potential, and drainage dynamics

Deep soil matric potential at 120 cm exhibited a consistent seasonal pattern across the study site during the March–October 2025 monitoring period. Matric potentials were generally between −20 to −30 kPa in March but rapidly transitioned to saturated conditions (0 kPa) as soils thawed and spring precipitation increased (Figure 4). From March through June 2025, deep soil matric potential for each plot remained near saturation (0 kPa), with brief drying intervals followed by rapid rewetting in response to precipitation events. Beginning in July 2025, deep soil matric potential became progressively more negative across most plots as corn growth and increasing atmospheric demand raised ET. Smaller and less frequent precipitation events during August and September 2025 contributed to further deep soil drying, whereas rainfall events near the October harvest partially rewetted the deep soil profile and increased soil matric potential. This seasonal pattern is consistent with prior work demonstrating positive soil water storage changes during late winter and spring, followed by net depletion during mid‐summer to late summer as ET increases (Brye et al., 2000). Intermittent gaps in matric potential data are present for several plots and reflect periods when individual control systems temporarily lost power.

**FIGURE 4:**
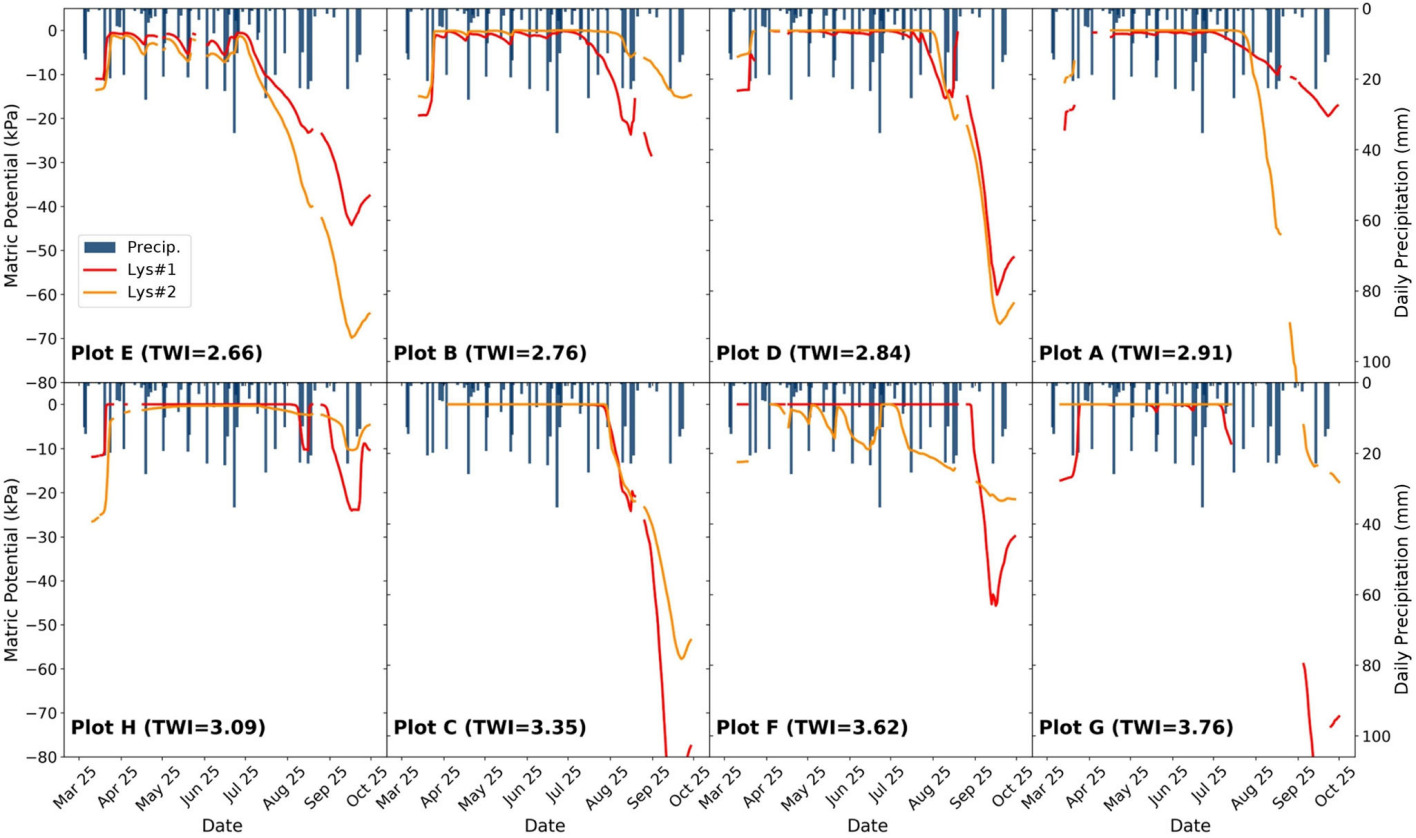
Average hourly soil matric potential (kPa) of soil adjacent to the lysimeter (Lys; 120‐cm depth), along with daily precipitation (mm), organized by increasing topographic wetness index (TWI).

Drainage collection closely followed seasonal changes in deep soil wetness. Measurable drainage below the root zone occurred primarily when soil matric potential at 120 cm approached saturation (Figure 5). Under these conditions, precipitation inputs exceeded available pore space, promoting deep percolation. In contrast, during mid‐summer to late summer, increasing ET and decreasing matric potentials increased the capacity of the soil profile to retain incoming precipitation, thereby limiting drainage despite occasional large precipitation events. As a result, cumulative drainage increased most rapidly in spring, prior to peak summer ET. This seasonal distribution of drainage is consistent with previous studies using equilibrium tension lysimeters in the Upper US Midwest, which reported that the majority of annual deep percolation occurs during late winter and spring, with comparatively little drainage collected during summer and fall (Brye et al., 2000; Masarik et al., 2014; Stenjem et al., 2019).

**FIGURE 5:**
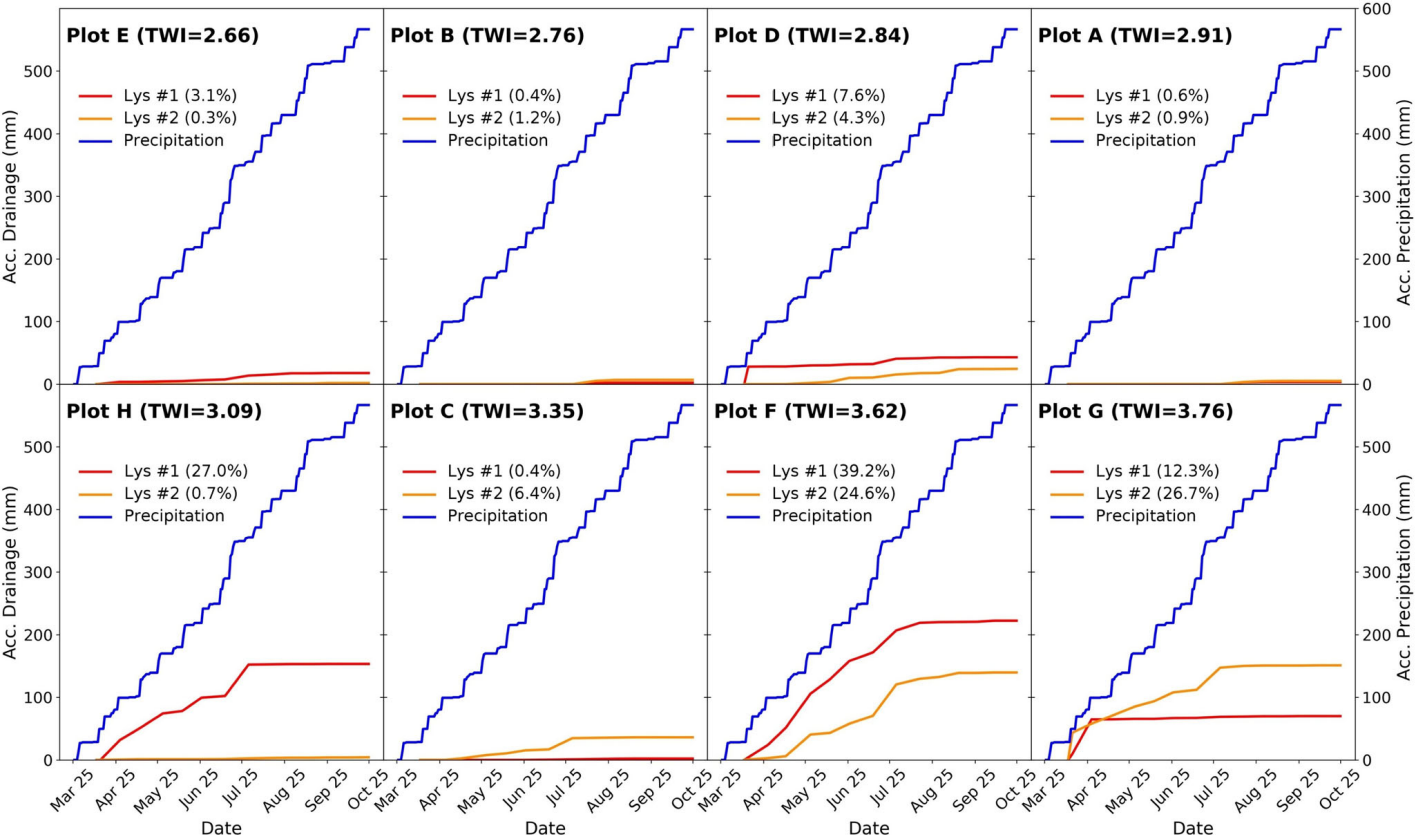
Cumulative precipitation (mm) and cumulative drainage (mm) collected from lysimeters organized by increasing topographic wetness index (TWI). Percentage of cumulative precipitation collected as cumulative drainage is shown in the individual graph keys.

To further evaluate lysimeter performance, the difference between applied lysimeter suction and the measured soil matric potential was quantified (Figure 6). The suction difference was defined as applied lysimeter suction minus measured bulk soil matric potential. The programmed suction offset defines a target operating range for this difference: between −2 kPa (suction differential) and −5 kPa (suction differential plus bleed differential). A suction difference within this range indicates that lysimeter pressure was maintained approximately 2–5 kPa more negative than the surrounding soil matric potential to establish a small hydraulic gradient toward the collection plate. This range should be interpreted as an approximate operational target rather than as a binary threshold of system performance, given uncertainty in both underlying measurements and the fact that matric potential was measured in bulk soil adjacent to the lysimeter rather than immediately above the collection plate (Supporting Information S1).

**FIGURE 6:**
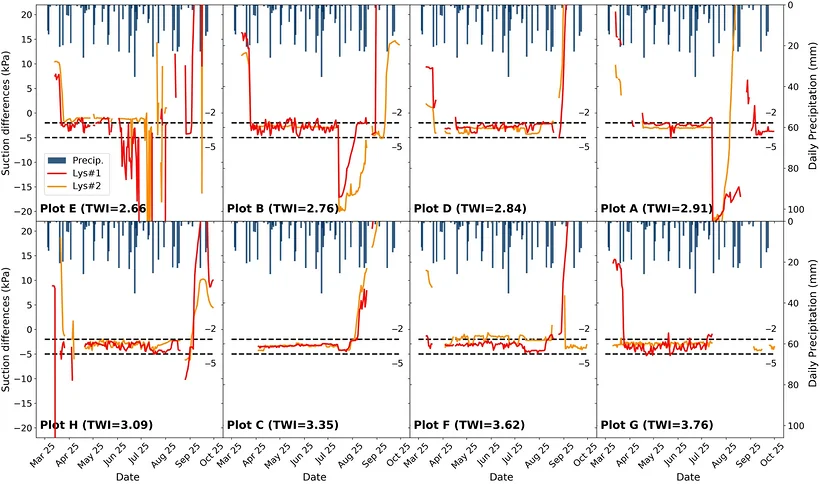
Average hourly suction difference (kPa) between the measured soil matric potential (120‐cm depth) and the lysimeter suction, along with daily precipitation (mm), organized by increasing topographic wetness index (TWI). The dotted lines represent −2 kPa (suction differential) and −5 kPa (suction plus bleed differential); a suction difference between −2 and −5 kPa suggests proper system performance.

The updated AETL performance exhibited clear seasonal trends that are critical for interpreting observed drainage variability. During March and early April 2025, suction differences were generally positive, or records were interrupted by battery outages, indicating that applied suction was insufficient relative to soil conditions (Figure 6) and that some lysimeters were not fully equilibrated during the early‐season thaw and wetting. From April through June 2025, suction differences for most lysimeters consistently fell within the target operating range, suggesting proper hydraulic coupling with the surrounding soil matrix during the period of greatest drainage. Beginning in July 2025, suction differences again became positive across many lysimeters as soil matric potential sharply declined under dry conditions, suggesting reduced hydraulic continuity between the soil matrix and the porous collection plate.

The sharp change in suction difference for lysimeters A1, A2, B1, and B2 at the end of June 2025 (Figure 6) resulted from deliberate manual intervention and troubleshooting. Because these lysimeters had collected little drainage up to June 2025, a suction differential of −20 kPa was applied for approximately 2 weeks to evaluate their ability to maintain hydraulic continuity and confirm that cavitation had not occurred. All four lysimeters successfully maintained the applied suction and produced small drainage volumes (<1 L) during this period (Figure 5), confirming system integrity.

The seasonal performance dynamics must be considered alongside spatial variability in deep soil moisture and drainage collection. Variability in soil matric potential among plots likely reflected a combination of small‐scale differences in microtopography, subsurface soil properties (structure and texture), and preferential flow. To contextualize the potential influence of microtopography on water redistribution, TWI values were calculated at the resolution of each plot pair (A‐H), with the two lysimeters in each pair assigned the same TWI value (Figures 4, 5, 6). TWI is a widely used index for characterizing the tendency of a landscape position to accumulate water based on upslope contributing area and local slope (Beven & Kirkby, 1979). Here, it was used as a qualitative interpretive framework rather than as a predictive model. Cumulative drainage generally increased with increasing TWI (Figure 5), suggesting that plot positions with greater potential for lateral water convergence more frequently reached the near‐saturation conditions favorable for deep percolation. However, cumulative drainage varied from less than 1% to nearly 40% of cumulative precipitation (Figure 5), indicating that drainage was controlled by multiple interacting factors rather than by plot position alone. Because paired lysimeters shared the same TWI value, TWI could not explain within plot pairs. These within‐pair differences were more consistent with variation in early‐season lysimeter performance (Figure 6), although small‐scale subsurface heterogeneity and preferential flow may also have contributed.

Incomplete equilibration of some lysimeters during March and April 2025 may have caused partial under‐collection of drainage during a hydrologically important part of the monitoring period, particularly for lysimeters with low cumulative drainage volumes. This timing is important because much of the annual drainage in US Midwestern agroecosystems occurs during late winter and spring (Brye et al., 2000; Masarik et al., 2014; Stenjem et al., 2019). In contrast, reduced lysimeter performance during July through September 2025 likely had minimal influence on cumulative drainage because deep soil matric potentials during this period were below field capacity (−10 to −33 kPa) and therefore the potential for deep percolation was limited.

Although the monitoring period did not capture late fall and winter drainage, several lysimeters produced cumulative seasonal drainage comparable to values reported in longer term AETL studies (Masarik et al., 2014; Stenjem et al., 2019). Lysimeters F1, F2, G1, G2, and H1 yielded substantially more drainage than others, whereas A1, A2, B1, and B2 produced markedly less. This variation likely reflected the combined influence of microtopographic redistribution, preferential flow pathways, subsurface heterogeneity, and early‐season differences in lysimeter performance. As noted by Masarik et al. (2014), spatial and interannual variability in drainage can be substantial and strongly modulated by precipitation magnitude and seasonal distribution. Continued plot‐scale monitoring will therefore be essential for distinguishing persistent landscape controls from early‐stage operational effects on deep percolation and associated solute transport.

### 3.2 Soil‐water budget

To provide an independent plausibility check on these measured drainage volumes, a simplified soil‐water budget was calculated for the monitoring period (March–October 2025). Because the estimate was sensitive to the parameterization of ET, drainage was bracketed under three soil‐water stress (Ks) assumptions: approximately 63 mm under unstressed conditions (Ks = 1), 136 mm using a wilting point water content of 0.15 m^3^ m^−3^, and 224 mm when stress was scaled between the observed minimum and maximum soil‐water contents during the monitoring period. The unstressed scenario (Ks = 1) produced the greatest estimated ET and therefore the lowest estimated drainage. Measured cumulative drainage from the better performing lysimeters (F1, F2, G1, G2, and H1) ranged from approximately 70 to 222 mm and fell within the water‐budget range, whereas the remaining lysimeters collected substantially less (generally <45 mm), below even the lowest drainage estimate of 63 mm. The consistency between the water‐budget range and the higher drainage volumes, together with the shortfall of the poorer performing lysimeters, supports the interpretation that at least part of the low measured drainage resulted from early‐season instrumental under‐collection (Figure 6) rather than from an absence of deep percolation at those locations. However, unquantified runoff and soil‐water storage changes may also have contributed to the observed variation. The soil‐water budget should therefore be interpreted as an order‐of‐magnitude consistency check rather than as an independent validation of drainage measured by individual lysimeters.

### 3.3 System shortcomings and improvements

Battery depletion represented the primary limitation of the AETL system during field deployment. Because system operation depends on continuous power to monitor soil matric potential and regulate lysimeter suction, battery failure results in a loss of equilibrium control and reduces the precision of AETL measurements. In cases of power failure, the AETL systems functioned at best like zero‐tension lysimeters, collecting only water arriving under saturated conditions. Multiple factors contributed to battery depletion, including reduced solar input and sustained high pump demand. Seasonal reductions in solar radiation during winter limited the ability of the solar array to recharge batteries, an effect exacerbated by consecutive overcast days. Under conditions where lysimeter suction could be maintained with minimal pumping, battery charge was generally stable. However, when lysimeter suction repeatedly failed to reach the target soil matric potential, the vacuum pump operated nearly continuously, rapidly depleting battery charge and leading to system shutdown until sufficient solar recharge occurred. Repeated deep discharge events further degraded battery performance, as lead‐acid batteries progressively lose charge‐holding capacity following multiple deep discharge cycles (Ruetschi, 2004). Higher capacity batteries and larger solar panels could partially mitigate deep discharge but would not address the underlying issue of sustained pumping when target suction cannot be achieved.

The sustained pumping responsible for these depletion events occurred primarily when lysimeters could not achieve or maintain the suction set point, which was particularly prevalent during late summer and winter when soils were relatively dry. This behavior was likely attributable to a loss of hydraulic continuity between the soil matrix and the lysimeter's porous collection plate as soil water content declined. As soils dry, unsaturated hydraulic conductivity decreases sharply and air‐filled porosity increases, such that water films connecting soil pores to the plate surface become thin and break. The resulting presence of air at the soil–plate interface reduces the effective area available for water transmission and prevents sustained vacuum application. This limitation is soil‐side, distinct from air entry into the porous collection plate or hydraulic resistance across it. Calculated pressure drop across the plate was negligible at all drainage fluxes observed or plausible at this depth, and air entry does not occur at the suctions applied by this system (Supporting Information S2). Similar limitations of equilibrium tension lysimeters under dry conditions have been noted previously (Brye et al., 1999).

The control logic imposed a maximum lysimeter suction threshold of −35 kPa (Section 2.3). When soils were drier than the threshold, lysimeters were often unable to reliably attain or maintain −35 kPa for the reasons described above. As a result, the system frequently entered prolonged pumping cycles without achieving equilibrium, highlighting the need for additional safeguards in the control logic beyond a fixed‐suction threshold. This limitation also implies that any drainage occurring at matric potentials below −35 kPa was not captured by the lysimeters, such that cumulative drainage measurements likely represent a modest underestimate of total drainage. However, because matric potentials below field capacity are associated with minimal vertical water movement, the magnitude of this underestimate is expected to be small.

An updated control logic was developed to address sustained pumping, preventing continuous pumping under unfavorable conditions while maintaining equilibrium behavior whenever possible (Figure 7, Supporting Information S3). This updated control logic reduces unproductive pump operation when hydraulic contact between the lysimeter porous collection plate and soil matrix is compromised, thereby conserving battery power and extending system uptime. These safeguards allow the system to operate within its physical limitations while protecting equipment and preserving data integrity. Importantly, prior studies (Brye et al., 2000; Masarik et al., 2014; Stenjem et al., 2019) and observations from this study indicate that most drainage collection occurred during spring and early summer, when deep soil layers were near saturation and lysimeters could reliably maintain suction. This updated logic was implemented in early 2026, after the monitoring period reported here; its effect on system performance across a full seasonal cycle, including the dry conditions that drove the original failures, remains to be evaluated.

**FIGURE 7:**
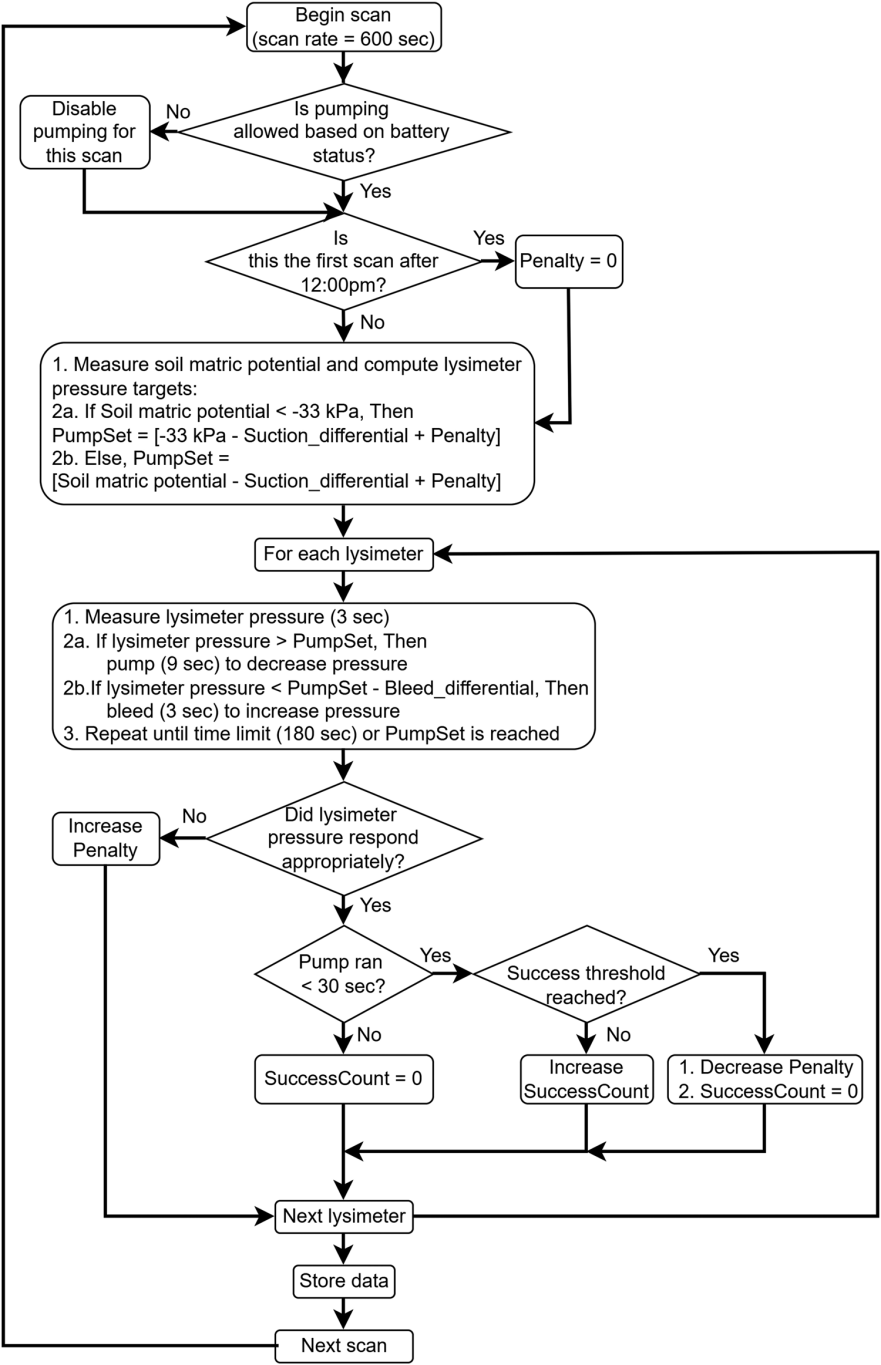
Conceptual flowchart of the lysimeter control algorithm used to maintain equilibrium between lysimeter suction and surrounding soil matric potential. The flowchart illustrates the primary decision pathways and feedback structure; detailed implementation, timing, and safety logic are provided in the Supporting Information.

### 3.4 Recommendations for future AETL design and implementation

These findings highlight several practical considerations for the design, installation, and interpretation of AETL networks in non‐tile‐drained agroecosystems. Reliable quantification requires attention to both instrumentation performance and landscape variability. During installation, firm contact between the soil matrix and the lysimeter porous collection plate is essential to minimize air gaps and prevent loss of suction. Systems should be installed, activated, and confirmed operational well before the intended measurement period. Throughout deployment, lysimeter suction should be monitored continuously alongside soil matric potential to diagnose system performance and confirm hydraulic coupling. Diagnostic adjustments to applied suction should be used sparingly, only when necessary to verify system integrity. Interpretation must account for the physical limits of the system. Because hydraulic continuity declines as soils dry, reduced late‐season collection should be interpreted as limited drainage potential rather than system failure. Adequate power capacity is also important for sustained operation; higher capacity batteries (i.e., 35 rather than 8 A‐h) and larger solar arrays can help maintain function during low‐recharge periods, particularly during winter. Landscape position warrants explicit attention in network design. Indices such as the TWI can guide placement, either normalizing lysimeters across topographic positions or deliberately stratifying them to capture drainage variability. Stratifying replication by landscape position, rather than by management unit alone, can improve interpretability without proportionally increasing instrument numbers.

## 4 CONCLUSIONS

This paper presents an updated AETL design and deployment framework integrating contemporary sensors, solar‐powered operation, remote communication, and revised control logic. One growing season of precipitation, deep soil matric potential, and cumulative drainage data documents both the system's behavior and its failure modes. Drainage at 120 cm was concentrated during near‐saturated conditions, consistent with prior Upper US Midwest studies. An independent soil‐water budget indicated that drainage collected by the higher yielding lysimeters was consistent with estimates derived from precipitation and ET, while lysimeters affected by early‐season equilibration and power limitations collected substantially less, indicating that instrumental performance, rather than an absence of drainage, accounted for much of the observed low collection. Remaining variability among lysimeters suggests small‐scale heterogeneity and microtopography may also influence drainage, although longer term datasets are needed to evaluate these effects.

While solute transport was beyond the scope of this manuscript, the AETL system is designed to support future studies examining how nutrient management practices interact with hydrologic conditions to influence solute movement below the root zone. Continued monitoring, paired with targeted solute analyses, will advance understanding of deep drainage and associated nutrient losses in non‐tile‐drained agricultural landscapes.

## AUTHOR CONTRIBUTIONS

**Mitch D. J. Wodrich**: Conceptualization; data curation; formal analysis; investigation; methodology; software; validation; visualization; writing—original draft; writing—review and editing. **Shuohao Cai**: Data curation; investigation; software; validation; visualization; writing—review and editing. **Kevin C. Masarik**: Methodology; software; writing—review and editing. **Jingyi Huang**: Conceptualization; methodology; resources; writing—review and editing. **Matthew D. Ruark**: Conceptualization; methodology; resources; writing—review and editing. **Xia Zhu‐Barker**: Conceptualization; funding acquisition; investigation; methodology; project administration; resources; supervision; validation; writing—review and editing.

## CONFLICT OF INTEREST STATEMENT

The authors declare no conflicts of interest.

## Supporting information



Supplemental Material S1 provides additional detail on the AETL system components, including tubing and fitting dimensions. Supplemental Material S2 expands on the sensor uncertainty analysis. Supplemental Material S3 presents the porous collection plate hydraulic resistance calculation. Supplemental Material S4 describes the updated AETL control logic; the control code (CRBasic) is available upon request.
