# Supplementary material for: Automated equilibrium tension lysimeter deployment to monitor vadose zone soil water and solute transport
Source: J Environ Qual. 2026 Oct 6;55(5):e70272. doi: 10.1002/jeq2.70272 (PMC13642970; doi:10.1002/jeq2.70272)
Supplement: Supplementary file 1 [file JEQ2-55-0-s001.docx]

**Automated Equilibrium Tension Lysimeter Deployment to Monitor Vadose Zone Soil Water and Solute Transport**

Mitch D.J. Wodrich^1^, Shuohao Cai^1^, Kevin C. Masarik^2^, Jingyi Huang^1^, Matthew D. Ruark^1^, Xia Zhu-Barker^1*^

^1^ Department of Soil and Environmental Sciences, University of Wisconsin-Madison, Madison, WI 53706, USA

^2^ College of Natural Resources, University of Wisconsin-Stevens Point, Stevens Point, WI 54481, USA

^*^Corresponding author: [zhubarker@wisc.edu](mailto:zhubarker@wisc.edu)

**Supplemental Material**

**Pages**: 4

**Sections**: 4

**Supplemental Material S1: AETL System Component Details**

All control system components were housed within a fiberglass enclosure measuring 41 cm × 46 cm (ENC 16/18; Campbell Scientific). Each stainless steel lysimeter vacuum port was connected to 6.2 mm (1/4 in) ID, 9.5 mm (3/8 in) OD polyethylene tubing using a compression fitting union (6-4 HBZ-SS; Parker Hannifin Corporation, Cleveland, OH), while the lysimeter sampling port was connected to 9.5 mm (3/8 in) ID, 12.7 mm (1/2 in) OD polyethylene tubing using a compression fitting union (8RU6-316; Parker Hannifin Corporation). Both vacuum and sampling lines were routed underground within PVC conduit (nominal 5.1 cm, Schedule 40) from the lysimeter to the control system enclosure. The PVC conduit extended approximately 100 cm above the soil surface adjacent to the control system enclosure with polyethylene tubing extending approximately an additional 30 cm. Sampling tubing was fitted with an airtight cap, while vacuum tubing was connected to the solenoid system using flexible PVC tubing with 3.1 mm (1/8 in) ID 6.2 mm (1/4 in) OD.

**Supplemental Material S2: Calculated Suction Difference and Sensor Uncertainty**

The suction difference was calculated as the difference between measured lysimeter pressure and the measured adjacent bulk soil matric potential. This range should be interpreted as an approximate operational target rather than as a binary threshold of system performance for the following reasons. First, the suction difference combines two independent measurements, each with associated uncertainty. The Teros 21 Gen 2 sensors have a manufacturer-reported accuracy of ±(10% of the reading + 2 kPa), corresponding to approximately ±2.0 kPa near saturation and ±5.5 kPa at -35 kPa. This uncertainty is comparable to, or greater than, the width of the operational target range. Lysimeter-pressure uncertainty was smaller because transducer offset drift was continuously corrected through in situ zeroing during each measurement cycle (Section 2.3), leaving a relatively small multiplicative span error at the low pressures observed during the monitoring period. Uncertainty in the calculated suction difference was therefore dominated by the matric potential measurement. Notably, because the reading-proportional component of this uncertainty was smallest near saturation, confidence in the suction difference was greatest during spring, when most drainage was collected. Individual observations should therefore not be interpreted as falling definitively inside or outside the operational target range.

Second, matric potential was measured in bulk soil adjacent to the lysimeter rather than immediately above the porous collection plate. Regulation of lysimeter pressure does not by itself demonstrate that the same pressure is established at the soil–plate interface, and no sensor was installed at that position in the present system. The calculated suction difference therefore indicates how closely lysimeter pressure approximated the matric potential of the bulk soil. Installing an additional matric potential sensor immediately above the plate, paired with the adjacent bulk soil sensor, could provide a more direct assessment of hydraulic coupling, though such an installation would disturb the soil in the zone whose undisturbed hydraulic behavior the lysimeter is intended to sample.

**Supplemental Material S3: Porous Collection Plate Hydraulic Resistance**

Hydraulic resistance across the porous collection was evaluated to determine whether plate transmission capacity could have constrained drainage collection. Applying the manufacturer's published pressure-drop relationship for this media grade (0.2 μm pore size, K_L_ = 90),

$\Delta P\left[ \mathrm{psid} \right]=K_{L}\times Flux[gpm \mathrm{ft}^{-2}]\times Viscosity[cp]\times Thickness[in]$	(S1)

the calculated pressure drop across the plate is below 0.001 kPa at the mean seasonal drainage flux of the highest-collecting lysimeter (approximately 1 mm d^-1^) and remains below 0.05 kPa at a flux of 100 mm d^-1^, which exceeds any plausible sustained deep drainage rate at 120 cm in these soils. The plate's thickness (approximately 1 mm, compared with the several-millimeter ceramic plates used in some tension lysimeter designs) and relatively large collection area (0.194 m^2^) contribute to this low resistance despite the fine nominal pore rating. Plate transmission capacity is therefore unlikely to limit collection, including during transient or preferential-flow events.

**Supplemental Material S4: Updated AETL Control Logic**

At the beginning of each scan, the program evaluates the battery status. If the battery voltage falls below a predefined threshold (12.3 V), a warning flag is triggered; if voltage declines to a critically low level (< 12.0 V), pumping is suspended while datalogging continues. Soil matric potential is then measured, and a target lysimeter pressure (PumpSet) is calculated as the measured soil matric potential minus a suction differential (2 kPa, included to establish a small hydraulic gradient driving drainage into the lysimeter) plus a dynamic penalty term (Penalty). If lysimeter pressure does not decrease meaningfully (>1 kPa) during a pumping cycle (180 sec), the attempt is classified as a failure and the penalty term increases. Under battery warning conditions (12.0–12.3 V), the penalty term increment is doubled (2 kPa rather than 1 kPa) to more rapidly relax the suction target and stabilize lysimeter pressure during low-power conditions. During subsequent scans, PumpSet is increased by the accumulated penalty value. This iterative adjustment continues until either a stable lysimeter pressure is achieved or PumpSet reaches 0 kPa. When meaningful pressure reduction (> 1 kPa) occurs during a pumping cycle, normal operation continues. To allow gradual re-tightening toward the measured soil matric potential, a success counter tracks cycles in which pump operation is minimal (< 30 sec). After a success threshold is reached (SuccessCount > 5), the penalty is reduced, resulting in a lower PumpSet during the next scan. If pumping exceeds minimal operation (> 30 sec) during any cycle, SuccessCount is reset. This adaptive process continues until the target pressure can be maintained without continuous pumping. The penalty counter is reset daily at noon. Finally, a bleed differential (3 kPa) defines the upper suction limit. If lysimeter suction exceeds this threshold (i.e., pressure becomes much lower than PumpSet), the system briefly vents the lysimeter to the atmosphere to restore the target range.
